# Case Report: *BMPR1A* and *BARD1* variants: first case identified in a Colombian family with juvenile polyposis syndrome

**DOI:** 10.3389/fonc.2026.1812499

**Published:** 2026-08-13

**Authors:** John Jairo Suárez, Jennyfer Benavides, Andrea Catalina Rubio-Vargas, Paul C. Lott, Alejandro Vélez, Jorge Mario Castro, Antonio Huertas, Magdalena Echeverry de Polanco, Luis G. Carvajal-Carmona, Mabel Bohórquez

**Affiliations:** 1 Cytogenetics, Phylogeny and Population Evolution Group, Faculty of Sciences and Faculty of Health Sciences, University of Tolima, Ibagué, Colombia; 2 Genome Center, Department of Biochemistry and Molecular Medicine, School of Medicine, University of California, Davis, Davis, United States; 3 Pablo Tobón Uribe Hospital, Dinámica Laboratory, Medellín, Colombia; 4 Federico Lleras Acosta Hospital, Ibagué, Colombia; 5 National Cancer Institute, Bogotá, Colombia; 6 Medicine Program, Department of Health Sciences, Tolima University, Colombia, Ibagué, Colombia

**Keywords:** *BMPR1A* and *BARD1* genes, frameshift insertion, germline variants, juvenile polyposis syndrome, premature stop codon

## Abstract

**Introduction:**

Juvenile polyposis syndrome (JPS) is a rare autosomal-dominant disorder characterized by numerous juvenile hamartomatous polyps in the gastrointestinal tract (GIT), increasing cancer risk. Pathogenic and likely pathogenic (P/LP) variants in the *SMAD4* or *BMPR1A* genes are associated with JPS. Here, we present a case of JPS with variants in *BMPR1A* and *BARD1*.

**Case description:**

This case involves a 62-year-old Colombian woman with multiple colorectal polyps. Family history included gastric cancer in the mother and the paternal grandmother, and colorectal polyps in two sisters and one daughter.

**Results:**

Germline testing of the proband identified a novel heterozygous frameshift variant in exon 11 of *BMPR1A* (NM_004329.3:c.1259dup) and a heterozygous frameshift variant in exon 11 of *BARD1* (NM_000465.4:c.2229dup), both predicted to introduce premature stop codons. The variants were validated by Sanger sequencing and evaluated by KASP genotyping in available relatives. In the JPS family, the *BMPR1A* variant was detected in the affected daughter and was absent in the tested unaffected relatives, supporting a presumed *de novo* origin, although parental testing was not available. In an independent case of cecal adenocarcinoma identified among 18 unrelated index cases, parental testing confirmed the *de novo* origin of the *BMPR1A* variant. To our knowledge, these variants have not yet been described in Colombia.

**Conclusion:**

Germline genetic testing revealed for the first time the presence of a possible novel *de novo* pathogenic variant in *BMPR1A* in a family with JPS and a case with an adenocarcinoma and family history of cancer, which may partially explain the etiology of the syndrome and facilitate cancer risk management for the other family members. In addition, the likely pathogenic variant in *BARD1* identified in our proband may be either incidental or causal. This latter gene is associated with an increased risk of breast cancer; however, additional evidence is required to support the role of this gene in the risk of JPS or GIT cancer.

## 1 Introduction

Juvenile polyposis syndrome (JPS, OMIM #174900) is a rare autosomal-dominant condition affecting approximately 1 in 100,000 to 160,000 individuals (1). The main clinical feature is multiple juvenile-type hamartomatous polyps in the gastrointestinal tract (GIT), most commonly in the colorectum (98%), stomach (14%), duodenum (7%), and jejunum/ileum (7%) (2–4). In general, most polyps in patients with JPS are benign, typically pedunculated juvenile polyps without dysplasia, and usually appear during the first decade of life. However, patients with JPS may occasionally present with concomitant dysplasia and adenomatous polyps, which can lead to misdiagnosis of JPS as familial adenomatous polyposis (FAP) in clinical practice (5). Individuals with JPS have a cumulative risk of developing malignant tumors in the GIT ranging from 9% to 50%, with an average age at diagnosis of 43.9 years (6, 7). Clinical diagnostic criteria have been established, and a diagnosis can be made in the presence of at least one of the following: (1) five or more juvenile polyps of the colorectum; (2) multiple juvenile polyps throughout the GIT; and (3) any number of juvenile polyps in combination with a family history of JPS (8).

Approximately 75% of newly diagnosed JPS cases are inherited in an autosomal-dominant pattern, whereas the remaining cases are sporadic and lack a family history of polyps; their pathological phenotype may result from *de novo* pathogenic variants (5, 9, 10). Previous studies have reported that approximately 45%–60% of patients with a clinical diagnosis of JPS carry germline pathogenic/likely pathogenic (P/LP) variants in either the SMAD family member 4 (*SMAD4*, OMIM #600993) or bone morphogenetic protein receptor type 1A (*BMPR1A*, OMIM #601299) genes (1, 5). The *BMPR1A* gene encodes a type I serine–threonine kinase receptor, a member of the transforming growth factor β (TGF-β)-receptor superfamily, that mediates bone morphogenetic protein (BMP) intracellular signaling by activating *SMAD4* and regulates differentiation of colonic epithelial cells (11). Although these two genes play significant roles in the TGF-β/BMP pathway, the two JPS subtypes exhibit different phenotypic characteristics (12). Nearly 60% of patients with JPS carrying P/LP variants in *SMAD4* may present with upper gastrointestinal polyps and hereditary hemorrhagic telangiectasia (HHT), also known as Osler–Weber–Rendu syndrome or JPS/HHT syndrome, characterized by recurrent epistaxis, arteriovenous malformations (AVMs) primarily in the lungs, liver, and brain, as well as skin/mucosal telangiectasias (13). In contrast, patients with *BMPR1A* variants primarily present with colonic polyps and may also present with various non-JPS phenotypes without hamartomatous polyps, such as primary ovarian insufficiency, atrioventricular septal defect, and left ventricular noncompaction; however, the relationship between these phenotypes and *BMPR1A* alterations remains poorly understood (14).

Here, we describe a case with multiple colorectal and gastric polyps harboring variants in the *BMPR1A* and *BARD1* genes, identified by whole-exome sequencing (WES). Furthermore, we performed segregation analysis in 25 family members and in 18 unrelated probands from families with a history of HPS (14 cases) and in families with a history of colorectal cancer (CRC) or cancer in first-degree relatives (4 cases) to further characterize the genetic etiology of the disease.

### 1.1 Case presentation

This case involves a 62-year-old Colombian woman enrolled in a national CRC genetics study that included 1,228 patients diagnosed with CRC (15). At age 38, she underwent colonoscopy for gastrointestinal symptoms, which revealed multiple polyps measuring 0.2–2.0 cm throughout the descending colon and rectum. Histopathological examination of multiple biopsies identified tubular, tubulo-villous, and villous adenomas, as well as hyperplastic polyps. Several villous adenomas exhibited high-grade dysplasia. Subsequent upper gastrointestinal endoscopy revealed numerous tubular polyps involving both the proximal and distal stomach. Histopathological examination demonstrated intestinal metaplasia with necro-inflammatory activity in the antral gastric mucosa.

The patient had a remarkable family history suggestive of hereditary gastrointestinal polyposis. Her mother (II-4) died of gastric cancer at age 75, and her paternal grandmother (I-2) also died of gastric cancer at an unknown age. One sister (III-7) was diagnosed with a tubulovillous adenoma with low-grade dysplasia in the descending colon at age 58, while another sister (III-9) was diagnosed with colorectal polyps at age 63. In addition, her daughter (IV-4) developed multiple colorectal polyps at age 17, involving the entire colon and rectum. Given the early onset of extensive gastrointestinal polyposis, the histopathological findings, and the family history suggestive of an inherited cancer predisposition syndrome, the patient and available relatives were referred for comprehensive germline genetic evaluation.

## 2 Materials and methods

### 2.1 Ethical approval

This study was approved by the Ethics Committee of the University of Tolima and was conducted in accordance with the principles of the Declaration of Helsinki. Written informed consent for genetic analysis and publication was obtained from the unrelated probands and participating family members.

### 2.2 DNA extraction and quality assessment

Peripheral blood samples were collected from probands and participating relatives. Genomic DNA was extracted using the Maxwell^®^ 16 Blood DNA Purification Kit (Cat. #1011) following the manufacturer’s instructions. DNA concentration and purity were measured using the NanoDrop ND-1000 Spectrophotometer (Thermo Fisher Scientific) and the Qubit dsDNA HS Assay (Thermo Fisher Scientific, Carlsbad, CA, USA). DNA integrity was assessed by agarose gel electrophoresis.

### 2.3 Whole-exome sequencing

WES was performed by Macrogen Inc. (Seoul, Republic of Korea) in accordance with the provider’s standard protocols for library preparation, exome capture, sequencing, and primary bioinformatics processing. WES data analysis was conducted as previously described (16, 17). Briefly, genetic data processing and germline variant calling were performed according to the Genome Analysis Toolkit (GATK) Best Practices guidelines (18). Raw sequencing data were obtained in paired-end FASTQ format, and all reads were aligned to the human reference genome 38 (GRCh38) using the Burrows–Wheeler Alignment (BWA-MEM) program. Variants were called with HaplotypeCaller and annotated with ANNOVAR. Only variants annotated as loss of function (stop gain, stop loss, or frameshift insertion/deletion) were included, as were variants showing a deleterious splicing effect predicted by Human Splicing Finder, missense variants classified as P/LP in ClinVar, or those meeting the clinical significance criteria established by the American College of Medical Genetics and Genomics (ACMG) guidelines. The sequencing data were simultaneously analyzed for copy number variations (CNVs) using the latest available version of VarSome Clinical (ExomeDepth R package) (v.11.5.0) and FranKlin by genoox (AI algorithm Rainbow) (v.2023.5).

### 2.4 Variant validation and segregation analysis

The variants were validated by Sanger sequencing using primers designed to amplify exon 11 of the *BMPR1A* gene (forward, 5′-ATCTGGCCCCAAGGAGAA-3′; reverse, 5′-CATGGCATGCCTGTATCAAA-3′) and exon 11 of the *BARD1* gene (forward, 5′-AGGCTTGAGCCTCATCCTTA-3′; reverse, 5′-CAATGACTGGGCTCTCACAA-3′). Genotyping was performed using Kompetitive allele-specific (KASP) PCR on an Illumina^®^ Eco™ Real-Time PCR System (LGC Genomics, Berlin, Germany) in 25 family members, including three JPS cases (two sisters and one daughter) and 22 unaffected family members, and in 18 index cases from families with a history of HPS (14 cases) and with a family history of CRC or cancer in first-degree relatives (4 cases) who participated in our CRC genetics study. Primer sets were designed using PrimerPicker Lite (Kbioscience, v.0.27). Each KASP primer set consisted of three primers: two KASP forward primers (BMPR1A: F1 ATGTAGGGCTGGAAGTGGTTTTTG and F2 GATGTAGGGCTGGAAGTGGTTTTTT; BARD1: F1 GAACCCTCTCTGGGTGATAATTAC and F2 CGAACCCTCTCTGGGTGATAATTAA) and one common reverse primer (BMPR1A: R1 CCAAACGCTACATGGCTCCCGAA and BARD1: R1 GTATATCATCTATGAAGATTTGT). The forward primers were synthesized with standard KASP fluorescent tails: The F1 primer carried the 5′ FAM tail (5′-GAAGGTGACCAAGTTCATGCT-3′), while the F2 primer carried the 5′ VIC tail (5′-GAAGGTCGGAGTCAACGGATT-3′). Graphical results of allele discrimination (genotyping) were visualized using the Illumina Eco System software.

## 3 Results

**Figure 1:**
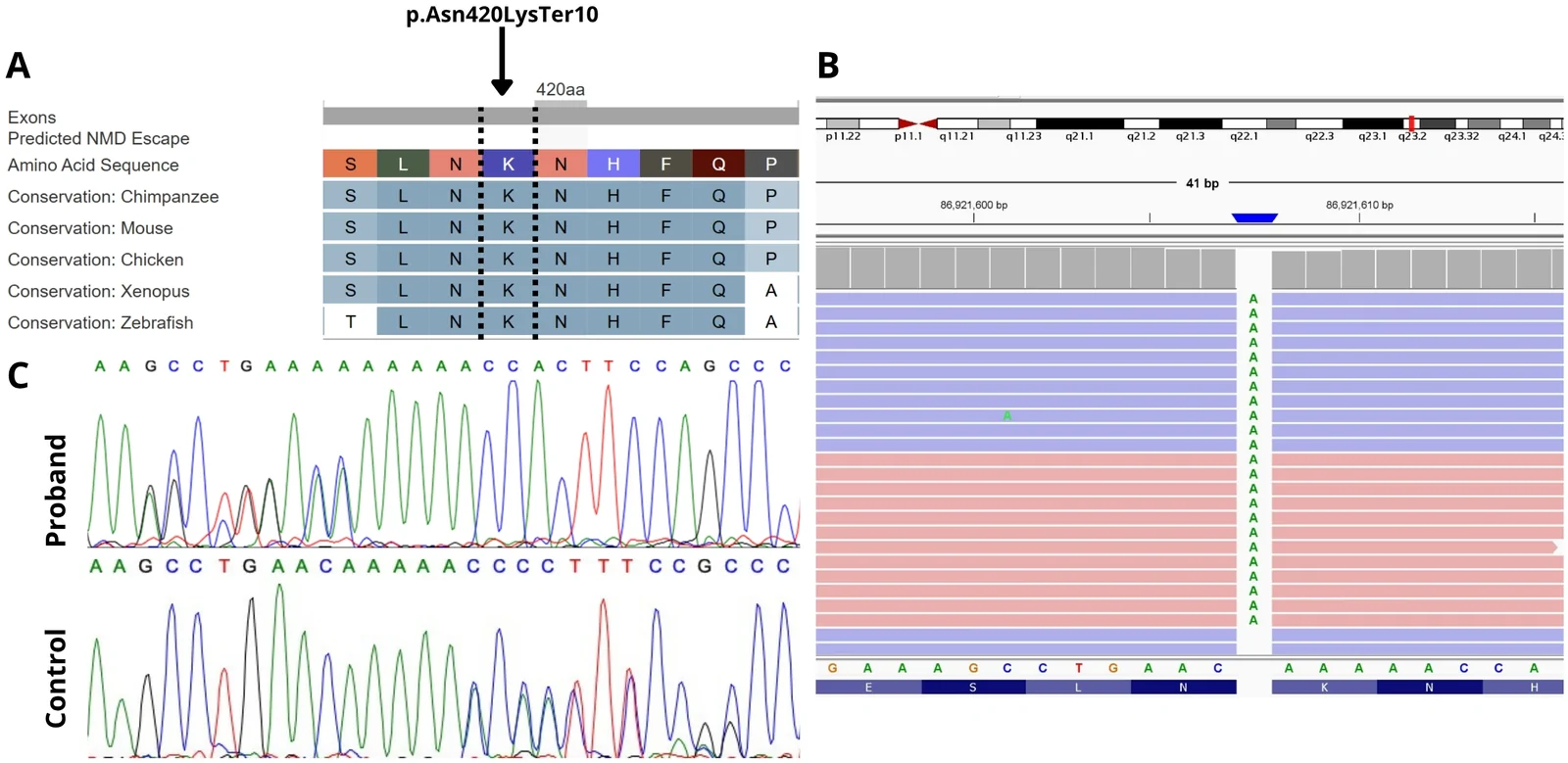
**(A)**. Evolutionary conservation analysis of the amino acid residue affected by the *BMPR1A* variant c.1259dup (p.Asn420LysfsTer10) across different species. Conservation data were obtained from www.Deciphergenomics.org **(B)**. Integrative Genomics Viewer (IGV) visualization of the frameshift insertion variant *BMPR1A* c.1259dup (chr10:86921607 C>CA). **(C)**. Sanger sequencing confirmed the insertion of an adenine (A) nucleotide corresponding to the c.1259dup variant.

**Figure 2:**
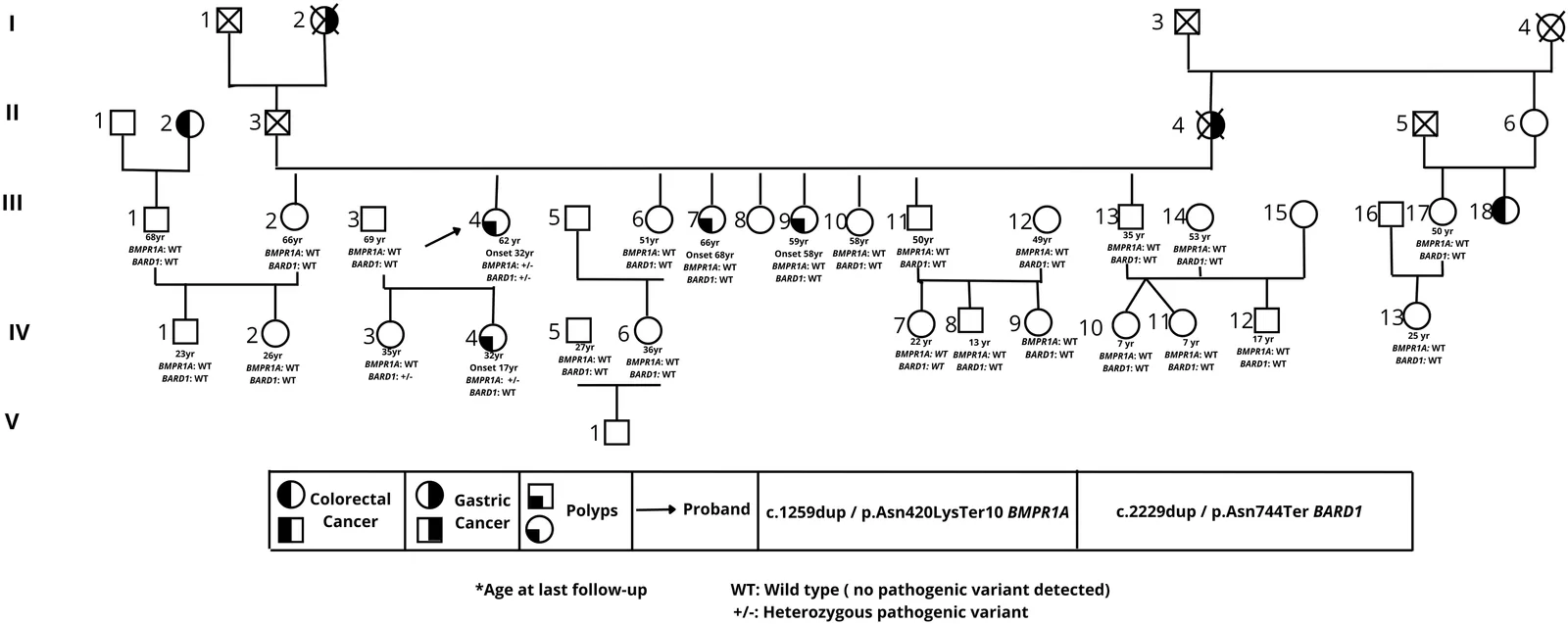
Pedigree of the family carrying variants *BMPR1A*. c.1259dup (p.Asn420LysTer10) and BARD1c.2229dup (p.Asn744Ter). Circles represent females and squares represent males. Individuals with available biological samples are indicated with the age at interview and age at disease onset below each symbol. Colorectal cancer (symbols with a middle-left quadrant), gastric cancer (symbols with a filled middle-right quadrant), and polyps (symbols with a filled lower-left quadrant). The proband is indicated by an arrow.

We identified a patient with a novel heterozygous frameshift insertion variant in exon 11 of the *BMPR1A* gene, resulting in a premature stop codon at amino acid position 430 (NM_004329.3:c.1259dup, p.Asn420LysTer10), as well as a heterozygous nonsense variant in exon 11 of the *BARD1* gene, causing a frameshift and premature stop codon at position 744 (NM_000465.4:c.2229dup, p.Asn744Ter) (**Figure 1**). The *BMPR1A* p.Asn420LysTer10 variant was carried by her daughter, who was diagnosed at age 17 with multiple colorectal polyps and gastritis (IV-4) (**Figure 2**). However, it was not found in her two sisters (III-7 and III-9), who were diagnosed with a colorectal polyp (adenoma). This variant has not been reported in the literature or in disease-related databases such as ClinVar, HGMD, or databases of mainly healthy individuals (gnomADv4.1.0, 1000GP). The amino acid position is highly conserved across species (PhyloP100way: 9.255), indicating that the site is important for normal protein function. According to the most recent ACMG guidelines, this variant is classified as pathogenic (class 5) based on the criteria PVS1 (AutoPVS1: Very Strong; frameshift variant, loss of function is a known disease mechanism, and it is predicted to escape NMD), PM6 (assumed *de novo*, with no parental confirmation available and phenotype highly specific for gene), and PM2 (absent or with no known minor allele frequency in the gnomAD population database). In addition, the variant was predicted to be deleterious by the online prediction tool ENTPRISE-X (Pathogenicity score 1). It was found to affect the kinase domain of the *BMPR1A* protein (amino acids 234–525) (9), resulting in an estimated 32% loss of the protein kinase domain.

**Figure 3:**
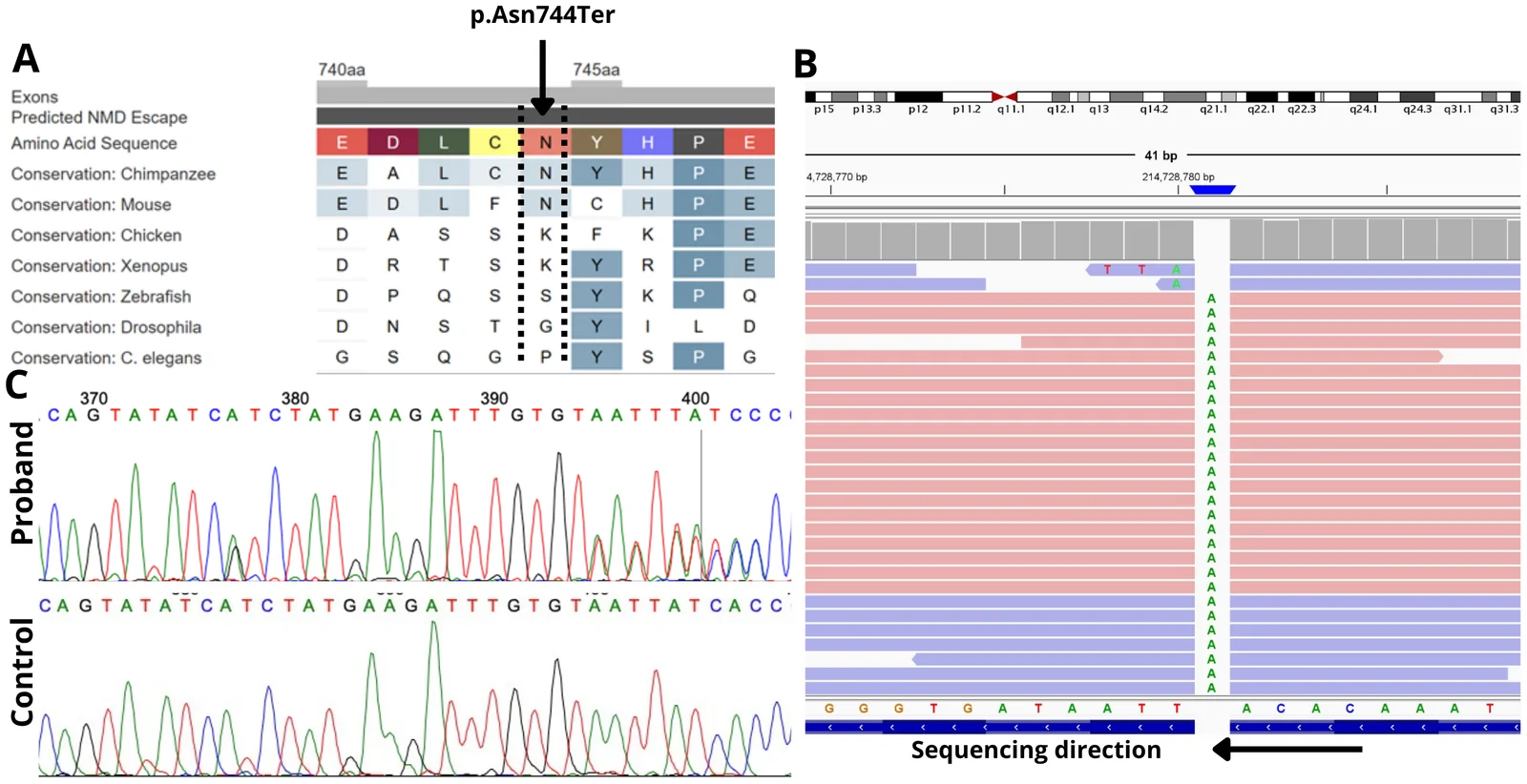
**(A)**. Evolutionary conservation analysis of the amino acid residue affected by the *BARD1* variant c.2229dup (p.Asn744Ter) across different species. Conservation data were obtained from www.Deciphergenomics.org **(B)**. Integrative Genomics Viewer (IGV) visualization of the insertion variant *BARD1* c.2229dup (chr2:214728780 T>TA). **(C)**. Sanger sequencing confirmed the insertion of an adenine (A) nucleotide corresponding to the c.2229dup variant. Electropherograms from the proband and a control individual are presented for comparison.

One healthy daughter was also found to carry the same nonsense *BARD1* variant, c.2229dup (p.Asn744Ter) (IV-3), which was absent in the remaining 24 family members (**Figure 2**). This variant causes a frameshift and a premature stop codon at the 3′ terminus of *BARD1*, resulting in the loss of the last 34 amino acids of the protein and affecting the BRCA1 C-terminal (BRCT2) domain of BARD1 (amino acids 667–777), which could result in a 30% loss of the protein’s BRCT2 domain. In addition, this variant was predicted to be deleterious by the online prediction tool ENTPRISE-X (Pathogenicity score 0.90). According to the ACMG guidelines (**Figure 3**), the variant fulfills the following criteria: PVS1 (AutoPVS1: strong, frameshift variant, loss of function is a known disease mechanism, and the truncated region is critical to protein function), PM6 (assumed *de novo*, with no parental confirmation available), and PM2 [the minor allele frequency is lower than 0.05% (0.00001666)]; therefore, we classified this variant as LP.

**Figure 4:**
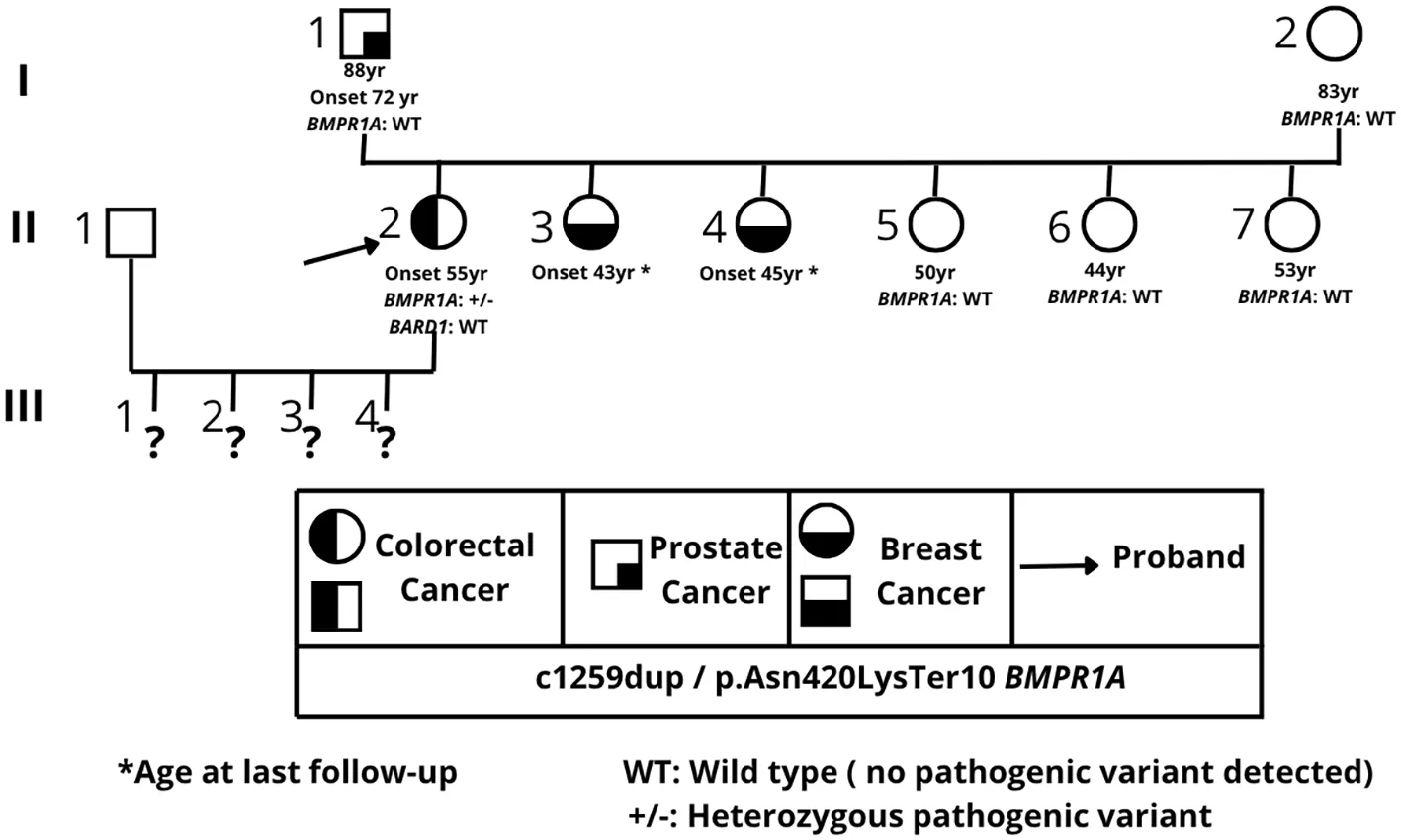
Pedigree of the family carrying the *BMPR1A* variant c.1259dup (p.Asn420LysTer10). Circles represent females and squares represent males. Individuals with available biological samples are indicated; age at interview (or last follow-up) and age at disease onset are shown below each symbol. An asterisk (*) indicates individuals for whom only family history information was available. Question marks (?) indicate relatives reported in the family history for whom no clinical, genetic, or follow-up information was available. Colorectal cancer is indicated by a filled middle-left quadrant, gastric cancer by a filled middle-right quadrant, and polyps by a filled lower-left quadrant. The proband is indicated by an arrow.

Finally, we identified the c.1259dup (p.Asn420LysTer10) variant in the *BMPR1A* gene in one patient among the 18 index cases in the CRC genetics study (**Figure 4**). The patient was a 55-year-old woman diagnosed with adenocarcinoma of the cecum, with a moderately differentiated exophytic pattern, involvement of the ileocecal valve, and a positive immunohistochemical evaluation of the mismatch repair system. Immunohistochemical evaluation showed retained nuclear expression of *MLH1*, *MSH2*, *MSH6*, and PMS2 in tumor cells, consistent with a mismatch repair-proficient (MMRp) phenotype. Her father (I-1) was diagnosed with prostate cancer at age 72, and two sisters were diagnosed with breast cancer at ages 43 (II-3) and 45 (III-4). The proband’s four children declined to participate in the study. Genetic testing of the proband’s father and three healthy sisters confirmed that the variant was *de novo*.

## 4 Discussion

JPS is a rare hereditary gastrointestinal polyposis syndrome characterized by variable clinical presentation and substantial genetic heterogeneity (1, 22). In this study, we describe a Colombian family with JPS carrying a novel presumed *de novo* pathogenic frameshift variant in *BMPR1A* (c.1259dup; p.Asn420LysTer10) together with a likely pathogenic variant in *BARD1* (c.2229dup; p.Asn744Ter). To our knowledge, this is the first report of this *BMPR1A* variant and one of the few reports of a presumed *de novo BMPR1A* alteration associated with JPS in Latin America. The clinical presentation, molecular findings, and segregation analyses expand the mutational spectrum of *BMPR1A*-associated disease and reinforce the value of comprehensive germline testing in patients with hereditary gastrointestinal polyposis.

The phenotype observed in this family is consistent with previously reported manifestations of *BMPR1A*-related JPS (19–21). The proband presented with extensive colorectal and gastric polyposis, including tubular, tubulo-villous, and villous adenomas, while her daughter developed multiple colorectal polyps during adolescence. The ages at diagnosis for both individuals are similar to those reported in previous studies of JPS, which often manifests during childhood or early adulthood (14). In contrast, relatives who lacked the familial *BMPR1A* variant exhibited either isolated adenomas or no evidence of gastrointestinal polyposis, supporting the pathogenic role of the identified variant and emphasizing the clinical utility of segregation analysis for distinguishing true hereditary disease from sporadic colorectal neoplasia.

Several observations support the interpretation that the *BMPR1A* variant is pathogenic and likely arose *de novo*. The variant is absent from major population and disease databases, affects a highly conserved residue, introduces a premature stop codon within the kinase domain, and fulfills ACMG criteria for pathogenicity. Furthermore, the variant co-segregated with the most severe polyposis phenotype within the family and was absent in the available unaffected relatives. Although parental DNA was unavailable for the JPS pedigree, additional evidence supporting a *de novo* origin came from an unrelated patient with CRC who carried the same variant, in whom testing of available relatives demonstrated that the alteration had arisen *de novo*. Together, these findings provide strong evidence that loss of *BMPR1A* function is the most plausible explanation for the gastrointestinal phenotype observed in this family.

Pathogenic variants in *BMPR1A* account for a substantial proportion of JPS cases and have also been associated with a broader spectrum of gastrointestinal phenotypes, including hereditary mixed polyposis syndrome, familial CRC, and early-onset MMRp CRC (14, 22). Consistent with these observations, we identified the same *BMPR1A* variant in an unrelated patient with cecal adenocarcinoma who did not meet the classical diagnostic criteria for JPS. This finding supports emerging evidence that constitutional *BMPR1A* alterations may exhibit variable expressivity and that not all carriers develop the classical hamartomatous polyposis phenotype. Consequently, the clinical spectrum associated with *BMPR1A* variants may be broader than currently appreciated and may include individuals presenting primarily with CRC or mixed colorectal neoplasia.

In addition to the pathogenic *BMPR1A* variant, we identified a likely pathogenic *BARD1* variant in the proband and one of her daughters. This alteration truncates the BRCT domain of the protein, a region involved in homologous recombination-mediated DNA repair and genomic stability (22, 23). Although *BARD1* is now recognized as a moderate-risk breast cancer susceptibility gene (8, 24), its role in gastrointestinal tumor predisposition remains uncertain (25, 26). Rare *BARD1* variants have previously been reported in families with CRC (27–29), but current evidence is insufficient to establish a causal relationship with JPS or gastrointestinal polyposis. Notably, the daughter carrying the *BARD1* variant did not carry the familial *BMPR1A* mutation and had no evidence of JPS at the time of evaluation, arguing against a major contribution of *BARD1* to the polyposis phenotype observed in this family. Therefore, the *BARD1* finding should currently be interpreted as an independent cancer-predisposition factor rather than a modifier of JPS, although future studies may help clarify its role in gastrointestinal cancer susceptibility.

This study has several limitations. First, the report is based on a single family, which limits conclusions regarding penetrance, genotype–phenotype correlations, and the clinical significance of the co-occurring *BARD1* variant. Second, parental samples were unavailable for the JPS pedigree, preventing definitive confirmation of the *de novo* origin of the *BMPR1A* variant in that family. Finally, functional studies were not performed; therefore, the biological consequences of the identified variants are inferred from computational predictions, segregation analyses, and existing knowledge of gene function. Additional studies involving larger cohorts and experimental validation will be required to better understand the mechanisms through which these variants contribute to gastrointestinal tumorigenesis.

In conclusion, we report the first Colombian family with JPS carrying a novel presumed *de novo* pathogenic *BMPR1A* frameshift variant together with a likely pathogenic *BARD1* variant. Our findings expand the mutational spectrum of *BMPR1A*-associated JPS, highlight the importance of segregation analysis for accurate diagnosis and genetic counseling, and suggest that the phenotypic consequences of *BMPR1A* alterations may extend beyond classical JPS. These results underscore the value of integrating detailed clinical characterization with comprehensive germline genetic testing to improve risk assessment, surveillance, and management of patients with hereditary gastrointestinal cancer predisposition syndromes.

## Data Availability

The original contributions presented in the study are included in the article/supplementary material. Further inquiries can be directed to the corresponding authors.
